# Tripartite motif-containing 27 negatively regulates NF-κB activation in bone remodeling

**DOI:** 10.1186/s10020-025-01204-7

**Published:** 2025-04-18

**Authors:** Kabsun Kim, Jung Ha Kim, Inyoung Kim, Semun Seong, Hyun Kook, Jeong-Tae Koh, Nacksung Kim

**Affiliations:** 1 Department of Pharmacology, Chonnam National University Medical School, Gwangju, 61469 Republic of Korea; 2 Hard-Tissue Biointerface Research Center, School of Dentistry, Chonnam National University, Gwangju, 61186 Republic of Korea; 3 Department of Pharmacology and Dental Therapeutics, School of Dentistry, Chonnam National University, Gwangju, 61186 Republic of Korea

**Keywords:** TRIM27, TAB2, Osteoclast, Osteoblast, NF-κB, Musculoskeletal disease

## Abstract

**Background:**

Tripartite motif-containing 27 (TRIM27) is highly expressed in the mouse thymus, spleen, and hematopoietic compartment cells and regulates cell proliferation, apoptosis, and innate immune responses. However, the role of TRIM27 in bone remodeling remains unknown. This study aimed to investigate the role of TRIM27 in the differentiation of osteoclasts and osteoblasts.

**Methods:**

We measured the effects of overexpression or knockdown of TRIM27 in osteoclasts and osteoblasts using real-time PCR and Western blot analysis to quantify the mRNA and protein levels of marker genes. Additionally, we performed an in vivo analysis of TRIM27 knockout mice through bone mineral density analysis and histological analysis.

**Results:**

TRIM27 deficiency decreased bone mineral density by enhancing osteoclast differentiation and inhibiting osteoblast differentiation. Overexpression of TRIM27 in osteoclast precursors suppressed osteoclast formation and resorption activity, and ectopic expression of TRIM27 in osteoblast precursors induced osteoblast differentiation and mineralization. Additionally, we found that TRIM27 attenuated NF-κB activation in both osteoclasts and osteoblasts by interacting with TAB2 and promoting TAB2 degradation through lysosomal-dependent pathways, thereby inhibiting NF-κB signaling.

**Conclusions:**

Our results identify TRIM27 as a novel negative regulator of NF-κB in bone remodeling, suggesting that regulating TRIM27 may be useful in developing treatments for musculoskeletal diseases, such as osteoporosis.

**Supplementary Information:**

The online version contains supplementary material available at 10.1186/s10020-025-01204-7.

## Background

Bone remodeling is continuous process orchestrated by osteoblasts, which form new bone, and osteoclasts, which resorb old bone. The balance between the osteoclast and osteoblast cells is crucial for maintaining skeletal integrity and preventing bone diseases such as osteoporosis, which reduces bone mass and increases fracture risk(Katagiri and Takahashi 2002). Osteoclast differentiation is stimulated by macrophage colony-stimulating factor (M-CSF) and receptor activator of NF-κB ligand (RANKL) (Boyle et al. 2003). RANKL, a member of the tumor necrosis factor (TNF) family, binds to its receptor RANK on osteoclast precursors, triggering a cascade of intracellular signaling, such as the mitogen-activated protein kinase (MAPK) pathway, the nuclear factor-κB (NF-κB) pathway, and the phosphoinositide 3-kinase (PI3K)/Akt pathway. This signaling cascade leads to the expression of genes associated with osteoclastogenesis, such as NFATc1, osteoclast associated receptor (OSCAR), and tartrate-resistant acid phosphatase (TRAP). Osteoblast differentiation is a tightly regulated process influenced by various growth factors and signaling molecules. Among these, Bone Morphogenetic Protein 2 (BMP2), a member of the transforming growth factor-beta (TGF-β) superfamily, initiates its signaling cascade by binding to specific BMP receptors on osteoprogenitor cells such as Wnt and MAPK pathways. This signaling promotes the commitment of cells to the osteoblast lineage by activating the transcription factor Runt-related transcription factor 2 (RUNX2), which regulates the expression of osteogenic genes such as alkaline phosphatase (ALP), bone sialoprotein (BSP), and osteocalcin (Lee et al. 2002).

Tripartite motif (TRIM) proteins are known to play significant roles in various biological processes, including cell proliferation, apoptosis, innate immunity, and inflammatory responses (Giraldo et al. 2020; Ma et al. 2016). (Hatakeyama 2017). TRIM proteins comprise a RING domain, one or two B-boxes, a coiled-coil domain, and a PRY–SPRY domain. TRIM proteins, which function as E3 ligases, are specified by the RING domain and ubiquitinate target proteins and regulate cellular signaling through protein degradation processes (Huang et al. 2022; Yang et al. 2020; Zhang et al. 2020). TRIM63 plays a significant role in bone remodeling by influencing both osteoblastic bone formation and osteoclastic bone resorption (Azuma et al. 2010; Kondo et al. 2011). TRIM65 has been shown to promote osteogenic differentiation by regulating the PI3K/AKT signaling pathway. TRIM21 deletion increases bone mass by enhancing osteogenic differentiation of bone marrow mesenchymal stem cells and reducing osteoclast formation (Liu et al. 2023). Additionally, TRIM38 acts as a negative regulator of NF-κB in bone remodeling by targeting TAB2 for degradation, thereby influencing both osteoblast and osteoclast activity (Kim et al. 2018). Although several reports have suggested a role for TRIM proteins in bone cells, various TRIM family members involved in bone homeostasis has not been studied. Therefore, we wondered whether other members of the TRIM family might also play a role in bone remodeling.

TRIM27 is expressed ubiquitously, although its expression levels may vary depending on the tissue type (Tezel et al. 2002). For example, TRIM27 is detected in mouse embryos from days 10.5–13.5, with its expression being restricted in adult mice (Cao et al. 1996). Additionally, high TRIM27 expression levels might be found in tissues with highly regenerative or a high turnover rate, such as the muscle, spleen, and thymus, indicating its potential role in cell proliferation and differentiation (Rajsbaum et al., 2008; Tezel et al. 1999; Tezel et al. 2002). In addition, TRIM27 expression has been reported to be upregulated in sciatic nerve transection-induced muscle atrophy.

TRIM27 can act as a RING-mediated E3 ubiquitin ligase to regulate signaling pathways by inducing the ubiquitination of other proteins, including Phosphatase and tensin homolog (PTEN), receptor-interacting protein 1 (RIP1), Janus Kinase 1 (JAK1), and TAK1 binding protein 2 (TAB2) (Chen et al. 2021; Conwell et al. 2015; Krutzfeldt et al. 2005; Nie et al. 2016; Shimono et al. 2000; Zaman et al. 2013; Zhang et al. 2018; Zhuang et al. 2016; Zurek et al. 2012). Further, TRIM27 negatively regulates skeletal muscle differentiation and may participate in the proteasome–ubiquitination pathway during skeletal muscle atrophy (Joung et al. 2014; Kee et al. 2012). Ubiquitin proteasome system was found to contribute to bone loss by regulating bone turnover and metabolism, by modulating osteoblast differentiation and bone formation as well as formation of osteoclasts that contribute to bone resorption (Lee et al. 2017). However, the role of TRIM27 in bone remodeling has yet to be elucidated.

In the present study, we investigated the role of TRIM27 in the differentiation of osteoclasts and osteoblasts, suggesting that TRIM27 functions as an E3 ligase to regulate NF-κB signaling by targeting TAB2 protein in bone cells. Our findings revealed a novel role of TRIM27 in bone remodeling, providing valuable insights into the molecular mechanisms underlying bone-related diseases and establishing the molecular basis for a potential therapeutic target in osteoporosis.

## Methods

### Mice

*Trim27*-deficient mice were obtained from Prof. Hyun Kook (Chonnam Nation University Medical School). The Chonnam National University Medical School Research Institutional Animal Care and Use Committee approved the experimental protocol, and the Chonnam National University Hospital Ethics Committee approved our experimental protocols (CNU IACUC-H-2021-60). All mouse-handling procedures and experiments were performed according to the National Institutes of Health guidelines (Guide for the Care and Use of Laboratory Animals).

### Cell culture media and reagents

Cell culture media and supplements were obtained from Hyclone. Recombinant human soluble receptor activator of nuclear factor kappa B ligand (sRANKL) was purified from bacteria, and recombinant human bone morphogenetic protein-2 (BMP-2) was purchased from Cowellmedi (#B5025, Busan, Korea). Alizarin Red S (#A5533), β-glycerophosphate (#G5422), and p-nitrophenyl phosphate (#7653) were purchased from Sigma-Aldrich (St. Louis, MO, USA). Ascorbic acid (#50-81-7) was purchased from Junsei Chemical (Tokyo, Japan).

### Retroviral gene transduction

Plat-E cells were maintained in Dulbecco’s modified Eagle medium (DMEM; HyClone Laboratories, Lagan, UT, USA) supplemented with 10% FBS. To prepare the retroviral supernatants, recombinant plasmids, and the parental pMX vector were transfected into the packaging cell line Plat-E using Fugene 6 (#E2692, Promega) according to the manufacturer’s protocol. Viral supernatant was collected from cultured media 48 h after transfection. BMMs or osteoblast precursor cells were incubated with the viral supernatants for 6 h in the presence of 10 µg/mL polybrene (#S2667, Sigma-Aldrich).

### Osteoclast differentiation, tartrate-resistant acid phosphatase staining, and pit formation

Murine osteoclasts were prepared from bone marrow cells as previously described(Kim et al. 2022). In brief, bone marrow cells were obtained by flushing femurs and tibiae from 6-week-old mice. Bone marrow cells were cultured in MEM-α containing 10% fetal bovine serum with macrophage colony-stimulating factor (M-CSF) (30 ng/mL) for 3 days, and attached cells were used as osteoclast precursors (BMMs). To generate osteoclasts, BMMs were cultured with M-CSF (30 ng/mL) and RANKL (100 ng/mL) for 3 days. All cells were cultured at 37 ℃ and 5% CO_2_. Cultured cells were fixed and stained for tartrate-resistant acid phosphatase (TRAP). TRAP-positive multinuclear cells containing more than three nuclei were counted as osteoclasts. Cells were observed using a Leica DMIRB microscope equipped with an N plan 10 × 0.25 numerical aperture objective lens (Leica Microsystems, Wetzlar, Germany). Images were obtained using a ProgRes CFscan (Jenoptik, Jena, Germany) camera and a ProgRes Capture Pro (Jenoptik). For the pit formation analysis, BMMs were cultured in Osteo assay plates (#3988, Corning, Corning, NY, USA) in the presence of M-CSF (30 ng/mL) and RANKL (100 ng/ml) for 4 days. Resorption lacunae were visualized under bright-field lighting using a Leica DMIRB microscope with a ProgRes CFscan (Jenoptik) camera and a ProgRes Capture Pro (Jenoptik).

### Osteoblast differentiation, alkaline phosphatase assay, and Alizarin red S staining

Primary osteoblast precursor cells were isolated from the calvarial bone of neonatal mice through enzymatic digestion using MEM-α containing 0.1% collagenase (#17018-029, Life technologies, Carlsbad, CA, USA) and 0.2% Dispase II (#12352200, Roche Diagnostics GmbH, Mannheim, Germany). After removing the enzymes, the collected cells were cultured in MEM-α containing 10% FBS, 100 U/mL penicillin, and 100 µg/mL streptomycin. Osteoblast differentiation was promoted by incubating in an osteogenic medium (OGM) containing 100 ng/mL BMP-2, 50 µg/mL ascorbic acid, and 100 µM β-glycerophosphate for 4 to 6 days, replacing the culture medium every 3 days. For the alkaline phosphatase (ALP) activity assay, osteoblast precursor cells were lysed using osteoblast lysis buffer (50 mM NaCl, pH 7.6, 150 mM NaCl, 0.1% Triton X-100, and 1 mM EDTA). The cell lysates were incubated with p-nitrophenyl phosphate substrate (#N7653, Sigma-Aldrich), and ALP activity was measured at 405 nm using a spectrophotometer. For Alizarin red staining, cells were cultured for 6 d, fixed with 70% ethanol, and stained with 40 mM Alizarin red (pH 4.2). After removing the nonspecific staining by washing with phosphate-buffered saline (PBS), Alizarin red staining was visualized using a CanoScan 4400 F (Canon Inc., Japan). Then, Alizarin red was dissolved in 10% cetylpyridium (#C0732, Sigma-Aldrich) at room temperature for 15 min, and Alizarin red activity was measured at 562 nm using a spectrophotometer.

### Quantitative real-time polymerase chain reaction

Cells were lysed in Qiazol (#79306, Qiagen GmbH, Hilden, Germany), and total RNA was isolated from the cultured cells according to the manufacturer’s protocol. Purified RNA was reverse transcribed into cDNA using a QuantiNova Reverse Transcription kit (#205411, Qiagen), including the procedure for removing any contaminating genomic DNA, according to the manufacturer’s instructions. The resultant cDNA was used for SYBR-based real-time PCR and performed in triplicate using a Rotor-Gene6 instrument (Qiagen). The thermal cycling conditions were as follows: 95 ℃ for 15 min, followed by 40 cycles of 95 ℃ for 15 s, 55 ℃ for 30 s, and 72 ℃ for 30 s. The mRNA concentrations were normalized to an endogenous housekeeping gene, *Gapdh*. The relative quantitation value for each target gene compared with the calibrator for the target was expressed as 2−(Ct − Cc) (Ct and Cc are the mean threshold cycle differences after normalizing to *Gapdh*). The relative expression levels of samples were presented using a semi-log plot. The sequences of used primers are listed in Table 1.

**Table 1:** The primer sequences of genes

Gene	Forward primer (5’-3’)	Reverse primer (5’-3’)
***Nfatc1***	CTCGAAAGACAGCACTGGAGCAT	CGGCTGCCTTCCGTCTCATAG
***Oscar***	TGCTGGTAACGGATCAGCTCCCCAGA	CCAAGGAGCCAGAACCTTCGAAACT
***Acp5***	CTGGAGTGCACGATGCCAGCGACA	TCCGTGCTCGGCGATGGACCAGA
***Trim27***	ACCTGCCCCGTGTGCCTTCAG	TACAGCTTCAGAGGCTCCCGGTG
***Alpl***	CAAGGATATCGACGTGATCATG	GTCAGTCAGGTTGTTCCGATTC
***Ibsp***	GGAAGAGGAGACTTCAAACGAA	CATCCACTTCTGCTTCTTCGTTC
***Bglap***	ATGAGGACCCTCTCTCTGCTCAC	ATGAGGACCCTCTCTCTGCTCAC
***Gapdh***	TGACCACAGTCCATGCCATCACTG	CAGGAGACAACCTGGTCCTCAGTG

### Plasmids and luciferase assay

pFlag-CMV-TRIM27-WT and pFlag-CMV-TRIM27-ΔR, which lack a RING domain, were obtained from Prof. Hyun Kook (Chonnam Nation University Medical School). TAB1 and 2 were obtained from Prof. Soo Young Lee (Ewha Womans University). The 293T cells were cultured in DMEM supplemented with 10% FBS. Cells were transfected with the indicated amounts of the expression plasmids using Fugene 6 (#E2692, Promega, Madison, WI), following the manufacturer’s protocol. After 48 h of transfection, the cells were washed twice with PBS and then lysed in reporter lysis buffer (#E4030, Promega). Luciferase activity was measured using the luciferase assay system (#E4030, Promega) according to the manufacturer’s instructions. Luciferase activity was measured in triplicate, averaged, and then normalized to β-galactosidase activity using ο-nitrophenyl-β-D-galactopyranoside (#48712-M, Sigma-Aldrich) as a substrate.

### Immunoprecipitation and Western blotting

Cells from transfected 293T cells, osteoclasts, or osteoblasts were harvested after washing with ice-cold PBS and lysed in extraction buffer (50 mM Tris–HCl, pH 8.0, 150 mM NaCl, 1 mM EDTA, 0.5% Nonidet P40, and 0.01% protease inhibitor cocktail). Samples immunoprecipitated with the indicated antibodies and whole cell lysates were subjected to SDS-PAGE and Western blotting. Primary antibodies used included TRAF6 (#sc-7221), NFATc1 (#sc-7294), Myc (#sc-42), Ub (#sc-8017, Santa Cruz Biotechnology, Dallas, TX, USA), IκB (#4812), p-IκB (#2859, Cell signaling technology, Danvers, MA, USA), OSCAR (#ab93817), ALP(#ab95462), Osterix (#ab22552), Osteocalcin (#ab93876, abcam, Cambridge, UK), TRIM27 (#MBS1492534, MyBioSource Inc. San Diego, CA), β-Actin (#A3854), HA(#A6533), and Flag (#A8592, Sigma-Aldrich). HRP-conjugated secondary antibodies (#ab6721, #ab6789, abcam) were used as probes and developed using an ECL solution (Millipore, Billerica, MA, USA). Signals were detected and analyzed by an Azure c300 chemiluminescent Western blot imaging system (Azure Biosystems Inc, Dublin, CA, USA).

### Micro-computed tomography and bone histological analyses

µCT images of distal femurs collected from 8-week-old mice were scanned using a high-resolution Skyscan 1172 system (Skyscan, Kontich, Belgium), which included an X-ray source at 50 kV and 201 mA with a 0.5 mm aluminum filter. Images were captured at 11 mm pixels, every 0.7° over an angular range of 180°. Raw images were reconstructed into serial cross-sections with identical thresholds (0 to 6000 Hounsfield units) for all samples using reconstitution software (CTAn, Skyscan). The trabecular bones at distances between 4.155 mm and 0.875 mm from the epiphyseal plate of distal femurs were manually designated as a region of interest (ROI), and femoral morphometric parameters were determined using µCT data analysis software (CTAn, Skyscan). Trabecular morphometry was characterized by measuring bone volume relative to tissue volume, trabecular thickness, trabecular number, and trabecular separation. For the three-dimensional visualization, pixels with thresholds between 90 and 165 were regarded as bones and processed using model visualization software (CTAn, Skyscan). For bone histomorphometric analysis, tibias collected from 8-week-old mice were fixed in 4% paraformaldehyde and decalcified in 5.5% EDTA buffer in 3.8% formaldehyde buffer at 4 °C for 2 weeks. The samples were gradually dehydrated, embedded in paraffin, and cut into 4 mm-thick longitudinal sections. After the sections were deparaffinized with xylene, they were stained with hematoxylin and eosin (H&E) or tartrate-resistant acid phosphatase (TRAP) to quantify osteoblasts and osteoclasts, respectively. Quantitative analysis showing the number of osteoclasts was performed by calculating the ratio of TRAP-stained surface to total bone surface. For immunohistochemistry analysis, paraffin-embedded sections were deparaffinized, rehydrated, immersed in retrieval solution (0.01 M citrate, pH6.0) and then heated in a steamer for 20 min. Endogenous peroxidase were quenched by incubating in 3% hydrogen peroxide for 20 min at room temperature. The slides were incubated overnight at 4 °C using anti-RUNX2 (#ab236639). After incubation with anti-rabbit specific IHC polymer detection kit HRP/DAB (#ab209101, abcam,) and washed with PBS, the sections were treated with 3,3’‑diaminobenzidine (Sigma‑Aldrich) at room temperature. The slides were imaged using an inverted microscope (Nikon). The number of RUNX2-positive osteoblasts was counted lining the trabecular bone surface.

### Statistical analysis

All experiments were independently repeated at least three times, and the *n*-value was the number of independent experiments or mice. All data are presented as the mean ± standard deviation (SD). All statistical analyses were performed using unpaired two-tailed Student *t*-test for comparison between two groups. Multiple comparisons were performed using one-way or two-way ANOVA. All statistical analyses were conducted with GraphPad Prism (version 10.0, GraphPad Software, La Jolla, CA, USA). A value of *p* < 0.05 was considered statistically significant.

## Results

### Trabecular bone mineral density is decreased in *Trim27*-deficient mice

**Fig. 1:**
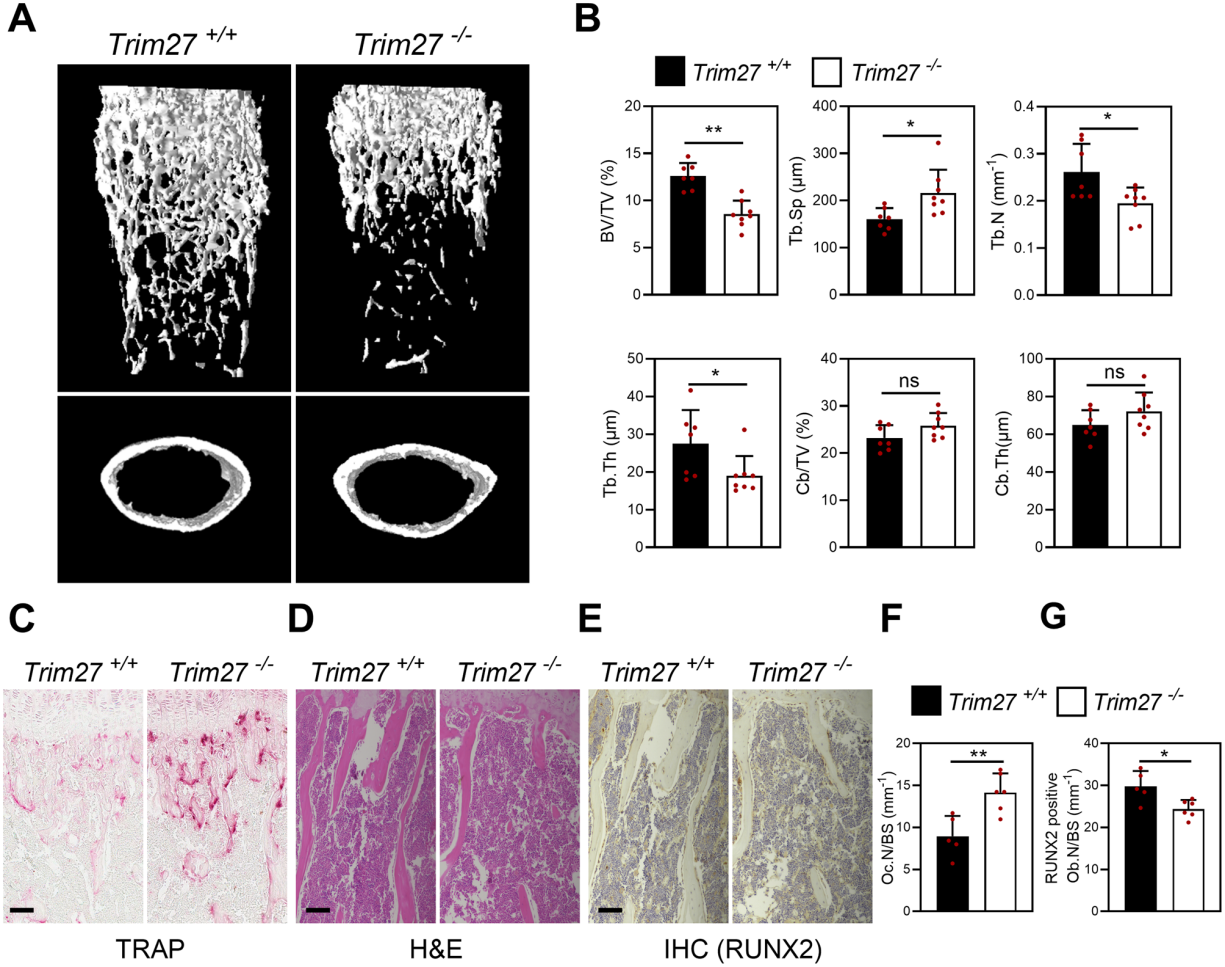
*Trim27* deficiency decreases trabecular bone mineral density. After mice were sacrificed, the long bones obtained from mice were subjected to µCT and immunohistochemical analyses. (**A**) Representative µCT images of trabecular and cortical bones in 8-week-old *Trim*27^+/+^ and *Trim*27^−/−^ mice. *Trim*27^+/+^, *n* = 7; *Trim*27^−/−^, *n* = 8. (**B**) Bone volume per tissue volume (BV/TV), trabecular separation (Tb.Sp), trabecular number (Tb.N), trabecular thickness (Tb.Th), cortical bone volume per tissue volume (Cb. BV/TV) and cortical bone thickness (Cb.Th) were assessed using the µCT measurements. *p < 0.05, **p < 0.01, vs. *Trim*27^+/+^. ns, not significant (*p* > 0.05). (**C**-**G**) TRAP staining (**C**), H&E (**D**) and IHC (RUNX2, **E**) of histological sections of proximal tibiae. Scale bar: 100 μm (**F**) Osteoclast number per bone surface per bone surface were assessed from histomorphometric analysis. (**G**) The number of RUNX2-positive osteoblasts lining the trabecular bone surface was counted. Data are presented as the mean ± SD, *n* = 3. *p < 0.05, **p < 0.01 vs. *Trim*27^+/+^

To investigate the physiological role of TRIM27 in bone in vivo, we analyzed the bone phenotype of the trabecular bone in the long bones from 8-week-old *Trim27* knockout mice using µCT imaging. *Trim27* knockout mice displayed reduced bone mass with decreased trabecular bone volume (BV/TV) and trabecular number (Tb.N), as well as increased trabecular bone separation (Tb.Sp) in the µCT analyses, compared to the wild-type group (Fig. 1A upper panel, and B). However, there was no difference in cortical bone volume between *Trim27* knockout mice and wild-type mice (Fig. 1A lower panel, and B). We conducted histological analyses to quantify osteoclasts and osteoblasts using TRAP stainin, immunohistochemistry for RUNX2, and H&E staining, respectively. In Trim27 knockout mice, we observed an increase in the osteoclast number per bone surface (Oc.N/BS) (Fig. 1C and F). Conversely, immunohistochemical analysis of RUNX2 and H&E staining revealed a decrease in the number of RUNX2-positive osteoblasts per bone surface (RUNX2-positive ob. N/BS) in Trim27 knockout mice compared to wild-type mice (Fig. 1D–E and G).

### *Trim27* deficiency enhances osteoclast differentiation

**Fig. 2:**
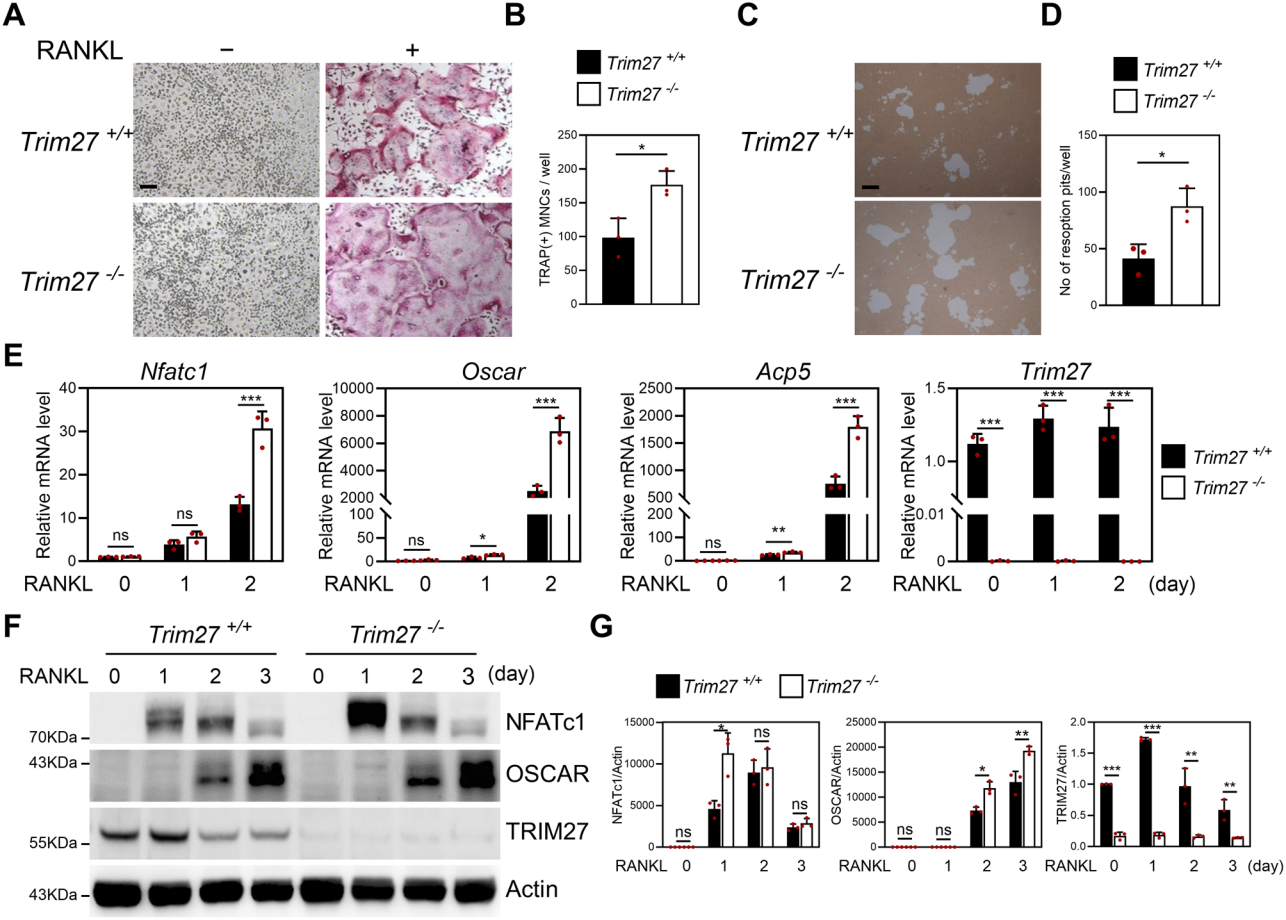
*Trim27* deficiency enhances RANKL-induced osteoclast differentiation. Bone-marrow-derived macrophage-like cells (BMMs) were harvested from long bones of *Trim27*^+/+^ or *Trim27*^−/−^ littermates. BMMs were cultured with M-CSF (30 ng/mL) either with or without RANKL (100 ng/mL) for 3 days. (**A**) Cultured cells were stained for TRAP. scale bar: 100 μm (**B**) The number of TRAP-positive MNCs per well was counted. *p < 0.05 vs. *Trim27*^+/+^. (**C**–**D**) BMMs were cultured on osteo assay plates for 4 days in the presence of M-CSF and RANKL. (**C**) Resorption lacunae were visualized by bright-field microscopy. scale bar: 100 μm (**D**) Numbers of resorption pits were counted; *p < 0.05 vs. *Trim27*^+/+^. (**E**) Total RNA was isolated from cell lysates, and qRT-PCR assessed *Nfatc1*, *Oscar*, *Acp5*, and *Trim27* mRNA expressions. Data represent the mean ± SD, *n* = 3. *p < 0.05, **p < 0.01, ***p < 0.001 vs. *Trim27*^+/+^. ns, not significant (*p* > 0.05). (**F**-**G**) Cells were harvested at the indicated time points, and WB lysates were analyzed using the specified antibodies. The protein levels of NFATc1, OSCAR, and TRIM27 were normalized to β-actin level (internal control). Data are presented as mean ± SD, *n* = 3; *p < 0.05, **p < 0.01, ***p < 0.001 vs. *Trim27*^+/+^. ns, not significant (*p* > 0.05)

To assess osteoclast differentiation in *Trim27*-deficient mice in vitro, we used bone marrow cells derived from *Trim27*-knockout mice. Cells were cultured in the presence of M-CSF and RANKL and stained for TRAP. We found that the number of TRAP-positive multinuclear cells (TRAP (+) MNCs) was increased in *Trim27*-deficient BMMs (Fig. 2A–B). Next, we investigated whether *Trim27*-deficient BMMs enhanced the formation of resorption pits using a synthetic calcium phosphate matrix. As shown in Fig. 2C and D, *Trim27*-deficient BMMs enhanced the formation of resorption pits compared to the wild-type BMMs. Then, we investigated whether *Trim27* deficiency regulates RANKL-induced osteoclast marker genes. After RANKL treatment, *Nuclear factor of activated T-cells cytoplasmic 1 (Nfatc1)*, a master regulator of osteoclastogenesis, was significantly increased, which induced the expression of osteoclast-specific genes *osteoclast-associated receptor (Oscar)* and *tartrate-resistant acid phosphatase (Trap/Acp5)* (Kim and Kim 2014). *Trim27* deficiency enhanced the expression of osteoclastic genes such as NFATc1, OSCAR, and TRAP as compared to wild-type cells during RANKL-induced osteoclast differentiation at both the mRNA levels (Fig. 2E) and protein levels (Fig. 2F−G).

### TRIM27 negatively regulates osteoclast differentiation

**Fig. 3:**
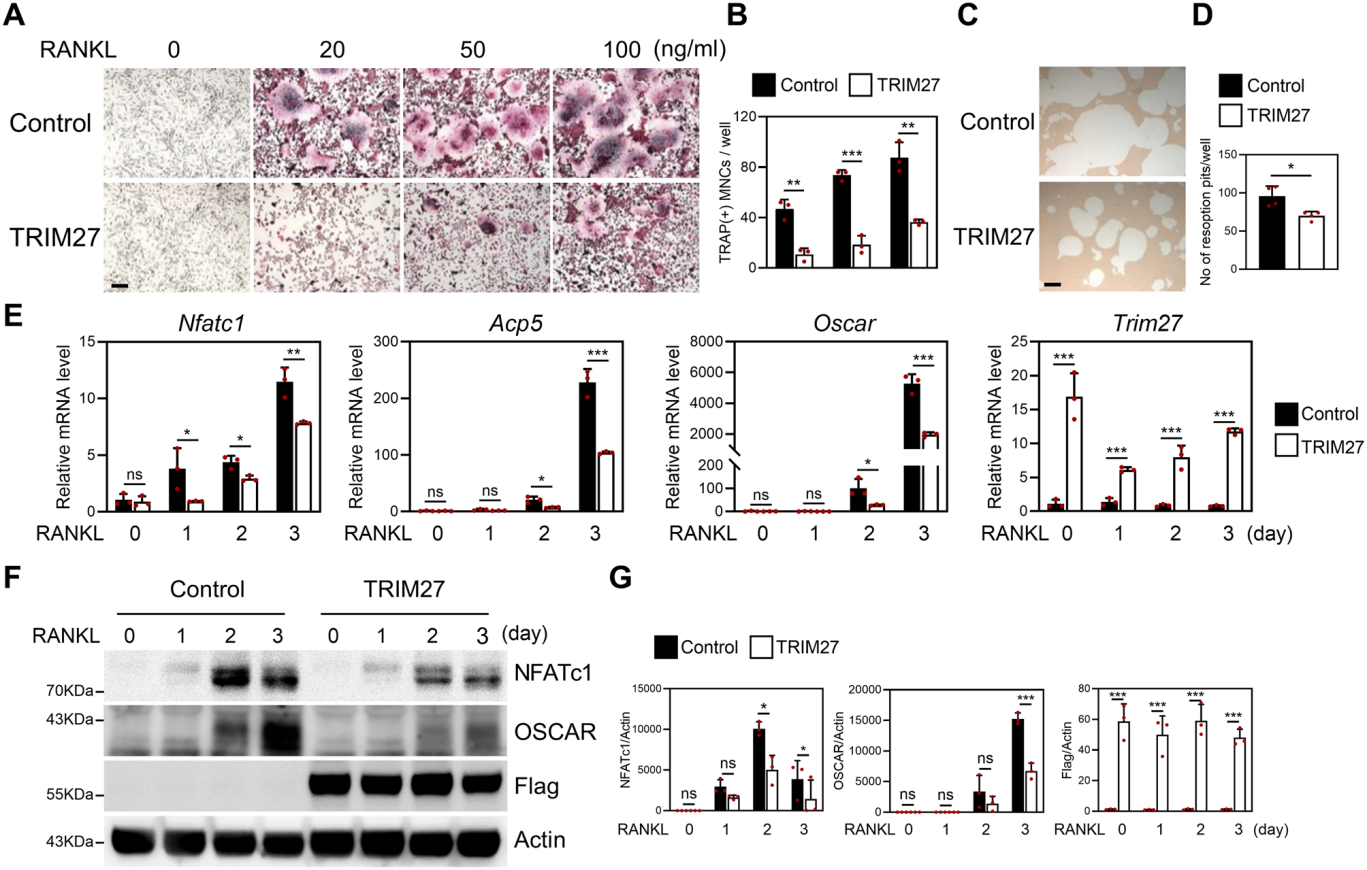
TRIM27 overexpression in BMMs suppresses RANKL-induced osteoclast differentiation. BMMs were infected with either pMX-IRES-EGFP (control) or TRIM27 retroviruses. (**A**) Transduced BMMs were cultured with M-CSF and various concentrations of RANKL for the indicated times. Cells were cultured for 3 days, then fixed and stained for TRAP. scale bar: 100 μm (**B**) The number of TRAP-positive multinucleated cells (TRAP (+) MNCs) per well was counted. **p < 0.01, ***p < 0.001 vs. the control. (**C**–**D**) Transduced BMMs were cultured on osteo assay plates with M-CSF and RANKL for 4 days. (**C**) Resorption lacunae were visualized using bright-field microscopy. scale bar: 100 μm (**D**) The number of resorption pits was counted. Data are presented as the mean ± SD, *n* = 3. **p < 0.01 vs. control. (**E**) Quantitative real-time PCR was performed to determine the mRNA expression of *Nfatc1*, *Oscar*, *Acp5*, and *Trim27*. Data represent the mean ± SD of triplicate samples. *p < 0.05, **p < 0.01, ***p < 0.001 vs. control. ns, not significant (*p* > 0.05). (**F**–**G**) Cells from each time point were harvested, and lysates were analyzed by Western blot using the specified antibodies. Protein levels were assessed using western blot. Data are presented as the mean ± SD, *n* = 3. **p < 0.01, ***p < 0.001 vs. control. ns, not significant (*p* > 0.05)

To explore the role of TRIM27 in osteoclast differentiation, a retroviral vector containing TRIM27 coding regions was constructed. Either the control or TRIM27 vector was overexpressed in BMMs using retroviral transduction. These transduced BMMs were cultured with various concentrations of RANKL in the presence of M-CSF. The number of TRAP-positive multinuclear cells (TRAP (+) MNCs) was increased following RANKL administration in a dose-dependent manner in BMMS infected with the control vector. However, TRIM27 overexpressing BMMs significantly inhibited RANKL-induced osteoclast formation (Fig. 3A–B). Next, to examine whether TRIM27 inhibited osteoclast functional activity, we investigated whether TRIM27 overexpression inhibits the formation of resorption pits on the surface using a synthetic calcium phosphate matrix. The formation of resorption pits was observed when transduced BMMs differentiated mature osteoclasts in the presence of RANKL. TRIM27 overexpressing BMMs strongly inhibited the formation of resorption pits compared to the controls (Fig. 3C–D). TRIM27 overexpression strongly attenuated RANKL-mediated induction of *Nfatc1*, *Oscar*, and *Acp5*, which are important for osteoclast differentiation, indicating that TRIM27 affects the expression of osteoclast marker genes at the mRNA level during osteoclast differentiation (Fig. 3E). Moreover, the protein expression of osteoclast factors, NFATc1 and OSCAR, were suppressed by TRIM27 overexpression (Fig. 3F−G). Based on these results, we suggest that TRIM27 negatively regulates RANKL-induced osteoclast differentiation and function.

### *Trim27* deficiency suppresses osteoblast differentiation

**Fig. 4:**
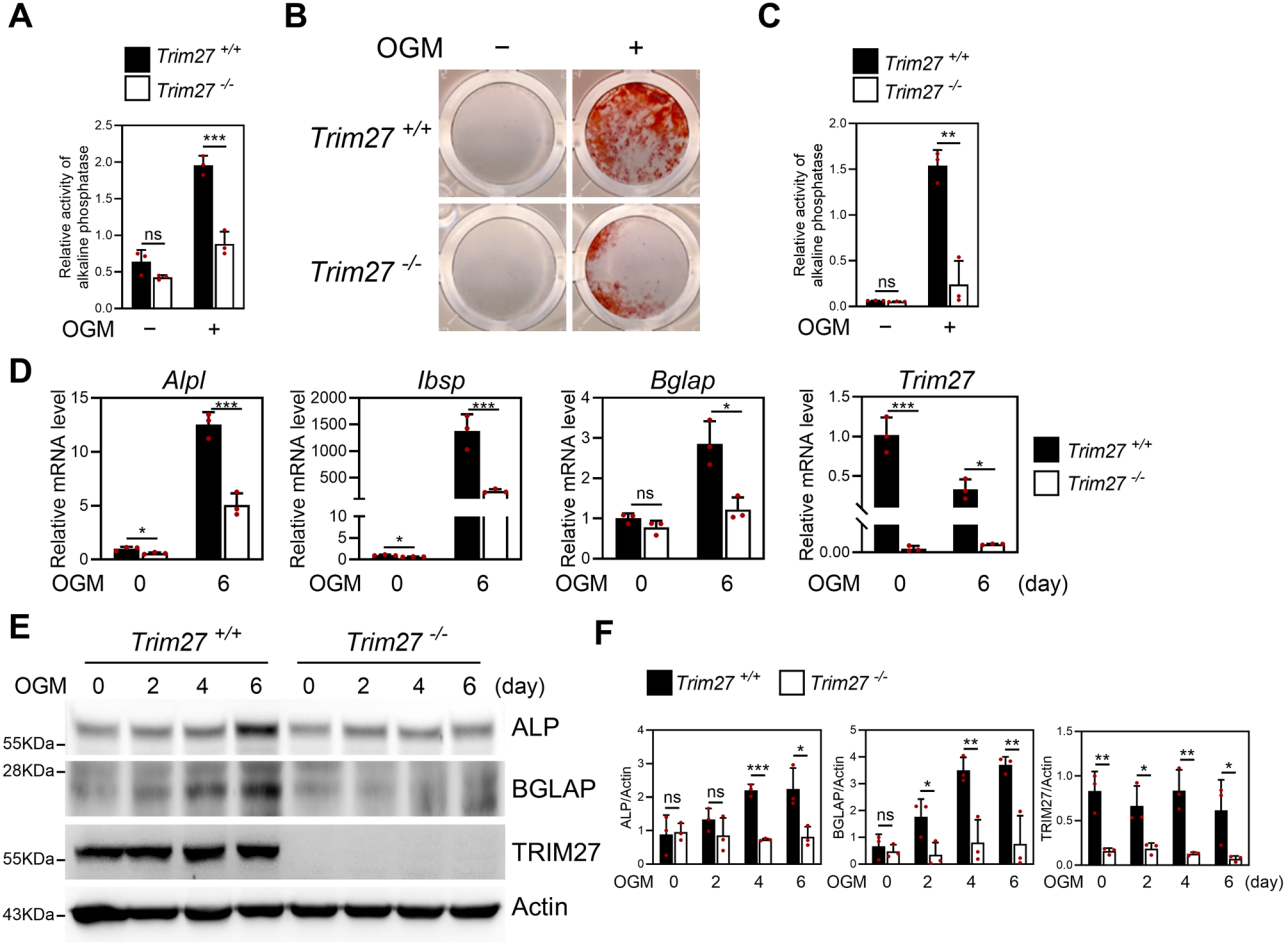
*Trim27* deficiency inhibits osteoblast differentiation. Primary osteoblast progenitor cells of *Trim27*^+/+^ or *Trim27*^−/−^ littermates were cultured in the presence of osteogenic medium containing BMP2 (100 ng/mL), ascorbic acid (50 µg/mL), and β-glycerophosphate (100 µM). (**A**) Cells were cultured for 4 days and analyzed for ALP activity. Data represent the mean ± SD of triplicate samples. ***p < 0.001 vs. *Trim27*^+/+^. ns, not significant (*p* > 0.05). (**B**) Cells were cultured for 6 days and fixed and stained with Alizarin red. (**C**) Alizarin red staining activity was quantified by densitometry at 570 nm. Data represent the mean ± SD, *n* = 3. **p < 0.01 vs. *Trim27*^+/+^. ns, not significant (*p* > 0.05). (**D**) Total RNA was isolated, and the mRNA expression levels of *Alpl*, *Ibsp*, *Bglap*, and *Trim27* were determined using quantitative real-time PCR. Data represent the mean ± SD of triplicate samples. *p < 0.05, ***p < 0.001 vs. *Trim27*^+/+^. ns, not significant (*p* > 0.05). (**E**-**F**) Cell lysates obtained at each time point were analyzed using Western blotting using the specified antibodies. The protein levels of ALP, BGLAP, and TRIM27 were normalized to β-actin level. Data are presented as mean ± SD, *n* = 3; *p < 0.05, **p < 0.01, ***p < 0.001 vs. *Trim27*^+/+^. ns, not significant (*p* > 0.05)

Next, to determine the effect of *Trim27* deficiency on osteoblast differentiation, we cultured primary osteoblast precursors obtained from calvariae of *Trim27*-deficient mice and wild-type littermates in the presence of osteogenic medium containing BMP-2, ascorbic acid, and β-glycerophosphate. We subjected these cells to an ALP activity assay and mineralized nodule staining to assess osteoblast differentiation and function, respectively. *Trim27* deficiency suppressed osteoblast differentiation as ALP activity (Fig. 4A), and Alizarin red staining was significantly decreased (Fig. 4B−C). During osteoblast differentiation, we observed a significant increase in the expression of osteogenic genes, including *Alpl*, *Bglap*, and *bone sialoprotein (Ibsp)*. However, the *Trim27* deficiency significantly inhibited the expression of these genes. In addition, we found that *Trim27* deficiency was accompanied by decreased mRNA and protein expressions of osteogenic marker genes required for osteoblast differentiation (Fig. 4D−F).

### TRIM27 overexpression enhances osteoblast differentiation

**Fig. 5:**
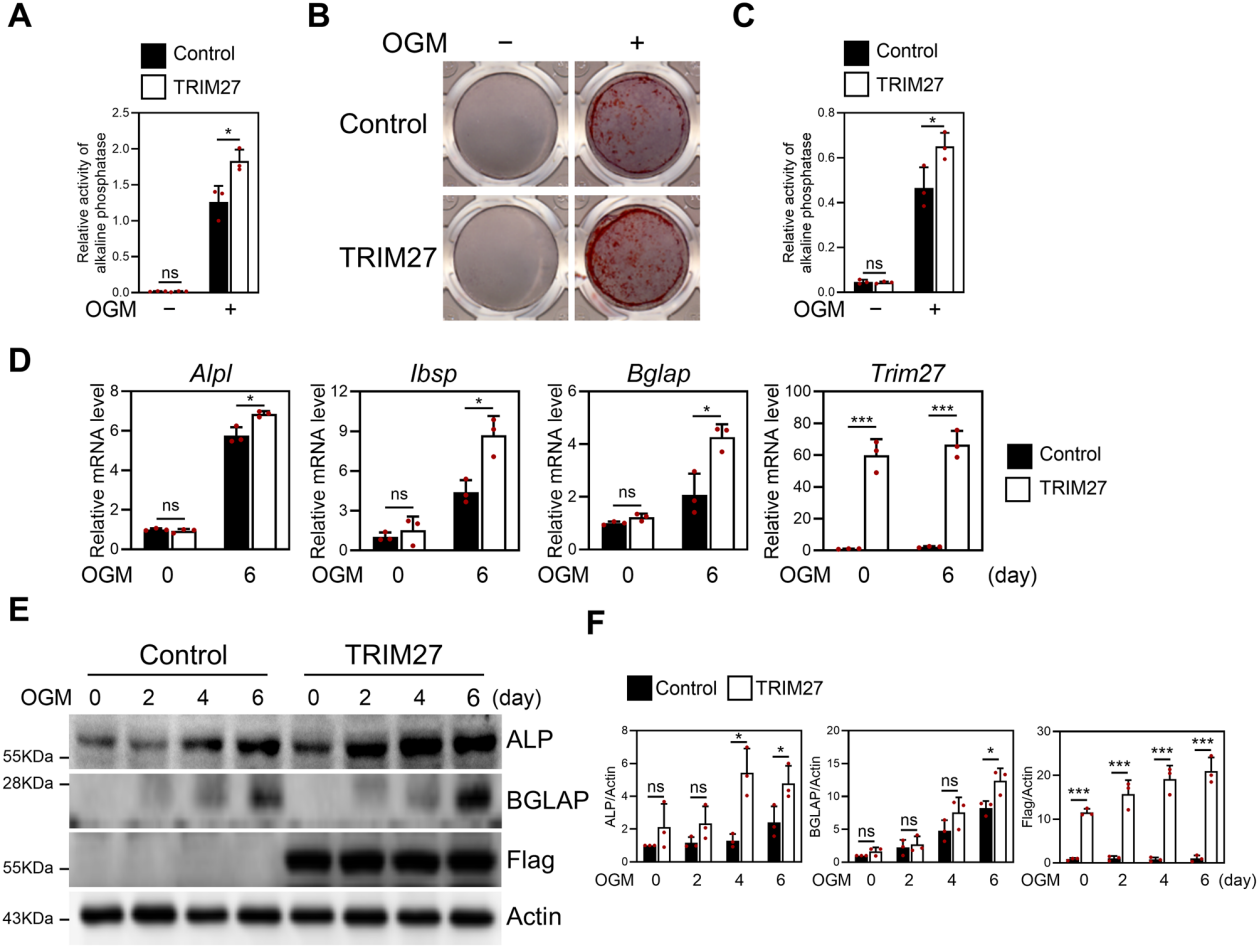
TRIM27 overexpression enhances osteoblast differentiation and function. Primary osteoblast precursor cells transduced with pMX-IRES-EGFP (control) or TRIM27 retrovirus were cultured in OGM. (**A**) Cells were cultured for 4 days and analyzed for ALP activity. Data represent the mean ± SD of triplicate samples. *p < 0.05, vs. control. ns, not significant (*p* > 0.05). (**B**) Cells were cultured for 6 days and fixed and stained with Alizarin red. (**C**) Alizarin red staining activity was quantified by densitometry at 570 nm. Data are presented as the mean ± SD, *n* = 3. *p < 0.05 vs. control. ns, not significant (*p* > 0.05). (**D**) Total RNA was isolated, and the mRNA expression levels of *Alpl*, *Bglap*, *Ibsp*, and *Trim27* were determined using quantitative real-time PCR. Data represent the mean ± SD of triplicate samples. *p < 0.05, ***p < 0.001 vs. control. (**E**–**F**) Cell lysates were obtained at each time point and analyzed by Western blotting using the specified antibodies. Protein levels were assessed through western blot. Data represent the mean ± SD of triplicate samples. *p < 0.05, ***p < 0.001 vs. control. ns, not significant (*p* > 0.05)

Given that TRIM27 is expressed in osteoblasts, we examined its role in osteoblast differentiation. Osteoblasts were infected with either the control or TRIM27 retrovirus and cultured in an osteogenic medium. We assessed ALP activity and bone nodule formation as osteoblast differentiation and function markers. Our results showed a significant increase in ALP activity in osteoblasts overexpressing TRIM27 osteoblasts compared to the controls (Fig. 5A). Similarly, TRIM27 overexpression in osteoblasts strongly enhanced mineralized nodule formation under osteogenic conditions (Fig. 5B−C). Furthermore, we observed that TRIM27 overexpression significantly enhanced the expression of *Alpl*, *Bglap*, and *Ibsp* during osteoblast differentiation (Fig. 5D). Moreover, TRIM27 overexpression increased the protein expression of osteoblast factors, such as ALP, and BGLAP (Fig. 5E−F). Overall, these results suggest that TRIM27 promotes osteoblastogenesis.

### TRIM27 regulates NF-κB activation in both osteoclasts and osteoblasts

**Fig. 6:**
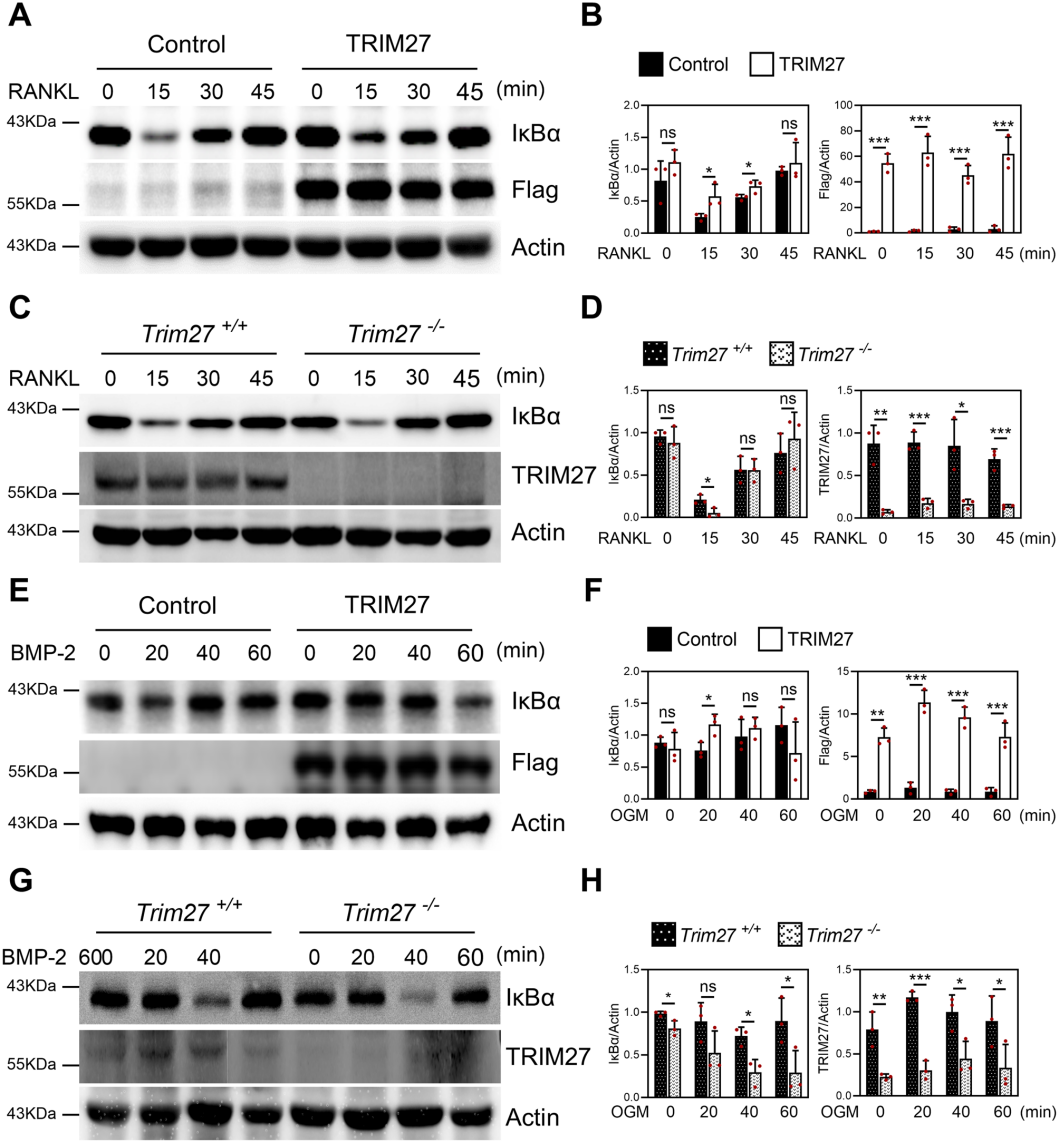
TRIM27 negatively regulates NF-κB activation. (**A**–**B**) BMMs were transduced with pMX-IRES-EGFP (control) or TRIM27 retrovirus and stimulated with RANKL for the indicated times. Whole-cell lysates were subjected to Western blotting using the indicated antibodies. The quantified IκBα protein level was normalized to β-actin level. Data are presented as mean ± SD, *n* = 3; *p < 0.05 vs. control. (**C**–**D**) BMMs were harvested from 6-week-old *Trim27*^+/+^ or *Trim27*^−/−^ and stimulated using RANKL for the indicated times. Whole-cell lysates were subjected to Western blotting using the indicated antibodies. Quantification of IκBα band intensity relative to β-actin was performed. Data are presented as mean ± SD, *n* = 3; *p < 0.05 vs. *Trim27*^+/+^. (**E**–**F**) Primary osteoblast precursor cells transduced with pMX-IRES-EGFP (control) or TRIM27 retroviruses were stimulated with BMP-2 for the indicated times. Whole-cell lysates were subjected to Western blotting using the indicated antibodies. Quantification of IκBα relative to β-actin was performed. Data are presented as mean ± SD, *n* = 3; *p < 0.05 vs. control. (**G**–**H**) Primary osteoblast progenitor cells of *Trim27*^+/+^ or *Trim27*^−/−^ littermates were stimulated with BMP-2 for the indicated times. Whole-cell lysates were subjected to Western blotting using the indicated antibodies. Quantification of IκBα relative to β-actin was performed. Data are presented as mean ± SD, *n* = 3; *p < 0.05 vs. *Trim27*^+/+^

TRIM proteins are well known for their regulatory role in the NF-κB activity (Tomar and Singh 2015). NF-κB is involved in inflammatory and immune responses and is important in regulating bone metabolism (Jimi and Katagiri 2022). We have previously reported that TRIM38 is important in differentiating osteoclasts and osteoblasts (Kim et al. 2018). Similarly, we determined whether TRIM27 regulates NF-κB activity in osteoclasts and osteoblasts. We first investigated whether TRIM27 regulates RANKL-induced NF-κB activation in osteoclasts. We introduced either control or TRIM27 retrovirus into BMMs and then stimulated them using RANKL. The control-vector-infected BMMs showed RANKL-induced degradation of IκBα. However, TRIM27 overexpression significantly blocked RANKL-induced IκBα degradation (Fig. 6A−B). We further explored whether *Trim27* deficiency regulates the NF-κB signaling pathway in contrast to TRIM27 overexpression in osteoclasts. Compared to the wild-type cells, *Trim27*-deficient cells showed changes in RANKL-induced IκBα degradation (Fig. 6C−D). Similar osteoblast experiments revealed that TRIM27 overexpression blocked BMP-2-induced degradation of IκBα (Fig. 6E−F). In contrast, *Trim27* deficiency had the opposite effect (Fig. 6G−H). Collectively, these data suggest that TRIM27 may inhibit the NF-κB signaling pathway in osteoclasts and osteoblasts.

### TRIM27 interacts with TAB2 and promotes TAB2 degradation through a lysosome-dependent pathway

**Fig. 7:**
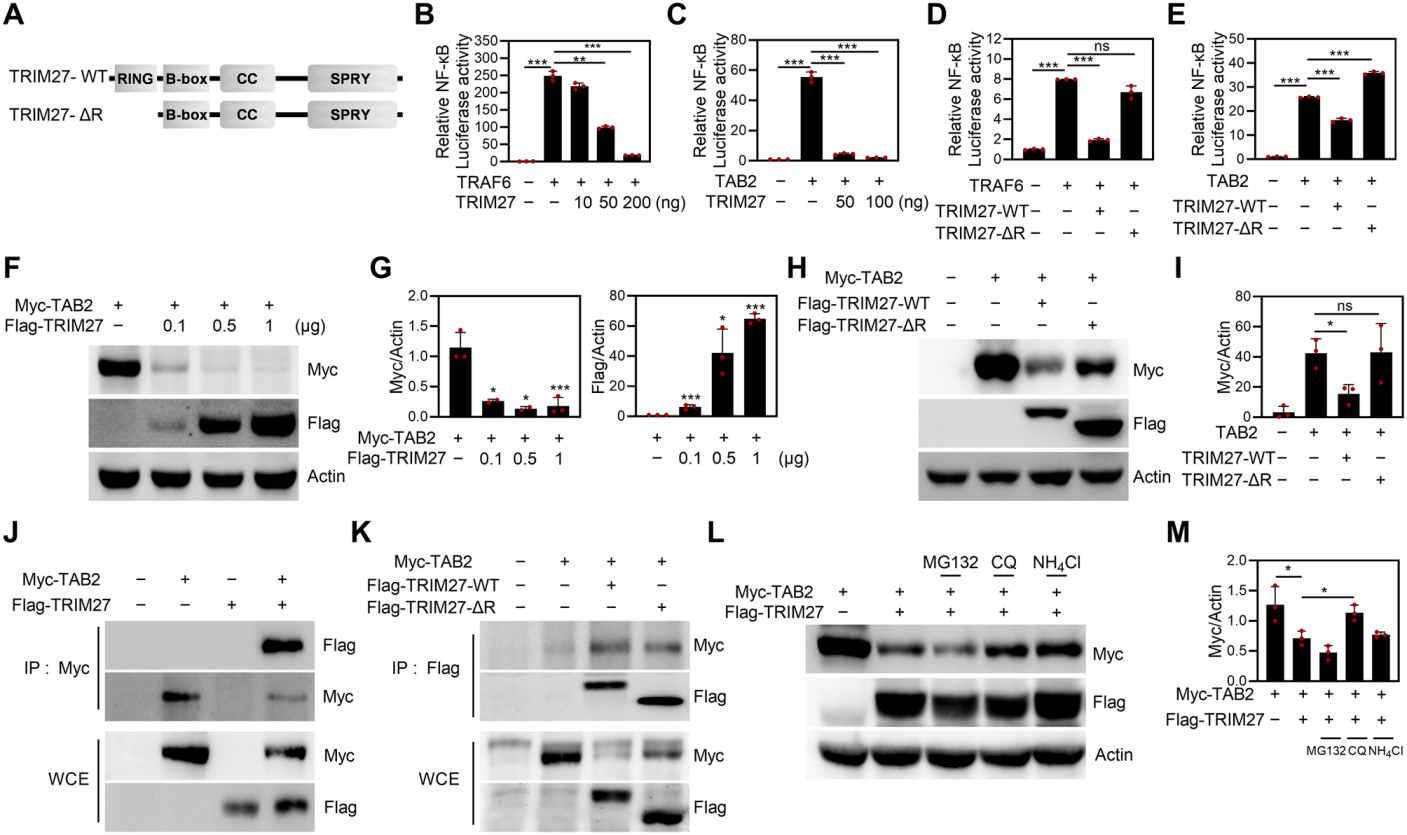
TRIM27 targets TAB2 and inhibits NF-κB activation through a lysosomal-dependent pathway. (**A**) Schematic representation of TRIM27-wild-type and TRIM27-deletion mutants. (**B**–**C**) 293T cells were co-transfected with the NF-κB luciferase reporter and TRAF6 (**B**) or TAB2 (**C**), as well as increasing concentrations of TRIM27. After 48 h of transfection, cells were lysed for luciferase assay. Luciferase activity was measured using a dual-luciferase reporter assay system. Data represent the mean ± SD of triplicate samples. **p < 0.01, ***p < 0.001 vs. control. (**D**–**E**) 293T cells were co-transfected with the NF-κB luciferase reporter and TRIM27-WT or TRIM27-ΔR with TRAF6 (**D**), or TRIM27-WT or TRIM27-ΔR with TAB2 (**E**). After 48 h of transfection, cells were lysed for the luciferase assay. Luciferase activity was measured using a dual-luciferase reporter assay system. Data represent the mean ± SD, *n* = 3. ***p < 0.001 vs. control. ns, not significant (*p* > 0.05). (**F**–**G**) 293T cells were transiently transfected with Myc-TAB2 and increasing amounts of Flag-TRIM27. After 48 h of transfection, cells were harvested and lysed. Cell lysates were subjected to Western blot analysis using the indicated antibodies. The protein levels of Myc and Flag were normalized to β-actin level. Data are presented as mean ± SD, *n* = 3; *p < 0.05, ***p < 0.001 vs. Myc-TAB2. (**H**–**I**) 293T cells were transiently transfected with TRIM27-WT or TRIM27-ΔR and TAB2. Cells were harvested and lysed. Cell lysates were subjected to Western blot analysis with the specified antibodies. The protein levels of Myc were normalized to β-actin level. Data are presented as mean ± SD, *n* = 3; *p < 0.05, vs. Mock. ns, not significant (*p* > 0.05). (**J**) 293T cells were cotransfected with Flag-TRIM27 and Myc-TAB2. Cell lysates were immunoprecipitated with an anti-Myc. The interaction between TRIM27 and TAB2 was analyzed by Western blotting using an anti-Flag or anti-Myc antibody. (**K**) 293T cells were transfected with Myc-TAB2 in the presence of Flag-TRIM27 or Flag-TRIM27-ΔR. Cell lysates were immunoprecipitated using anti-Flag. Coimmunoprecipitation and immunoblot analyses were performed using an anti-Flag or anti-Myc antibody. (**L**–**M**) 293T cells were transfected with either Myc-TAB2 or Myc-TAB2 with Flag-TRIM27. Before harvesting, cells were treated with or without MG132 (25 µM), CQ (100 µM), and NH_4_Cl (100 µM) for 4 h. Whole-cell lysates were analyzed by immunoblotting with a Flag or Myc antibody. The protein levels of Myc were normalized to β-actin level. Data are presented as mean ± SD, *n* = 3; *p < 0.05 vs. Myc-TAB2

TRIM proteins, which contain a RING domain, are part of a large family of E3 ubiquitin ligases and regulate cell signaling through proteolysis by ubiquitinating target proteins. Thus, to investigate the effect of the TRIM27 RING domain on NF-κB activation, we constructed a TRIM27 mutant by deleting the RING domain (TRIM27-ΔR) (Fig. 7A). Since we observed that TRIM27 inhibits the activation of NF-κB signaling in osteoclasts and osteoblasts, we further examined the effect of TRIM27 on NF-κB activation in 293T cells, mediated by various molecules, using luciferase reporter assays. We observed that TRAF6- or TAB2-induced NF-κB reporter activity was inhibited by the co-transfection of TRIM27 in a dose-dependent manner (Fig. 7B–C). We further assessed the effects of the TRIM27-ΔR mutant on the induction of NF-κB activity by TRAF6 or TAB2. Expectedly, TRIM27-ΔR failed to block TRAF6- or TAB2-induced NF-κB luciferase activity (Fig. 7D−E). Chen et al. reported that TRIM27 is a key regulator of hepatic I/R injury by mediating the degradation of TAB2/3. To assess whether the TAB2 protein level is regulated by TRIM27, 293T cells were transfected with TAB2, together with increasing amounts of the TRIM27 plasmid. Intriguingly, the TAB2 protein level decreased dose-dependently following the addition of TRIM27 (Fig. 7F−G). TRIM27 promoted TAB2 degradation, which was inhibited by TRIM27-ΔR (Fig. 7H−I). Next, we shifted our focus to TRIM27-driven TAB2 degradation, which is linked to its interaction. Thus, IP experiments were performed with Myc following the co-transfection of Flag-TRIM27 and Myc-TAB2 into 293T cells. Our results showed that TRIM27 interacts with TAB2 (Fig. 7J). We also examined whether the TRIM27 RING domains are responsible for the interaction with TAB2 coprecipitated with TRIM27-WT and TRIM27-ΔR. Interestingly, we observed that TRIM27 interacts with TAB2 irrespective of the RING domain (Fig. 7K). Since we observed that TRIM27 degraded TAB2 protein expression, we investigated how protein degradation is involved in TRIM27-mediated TAB2 degradation using various inhibitors. Therefore, TRIM27-mediated TAB2 degradation was blocked by the lysosomal inhibitors NH_4_Cl and chloroquine (CQ) but not by MG132, a proteasome pathway inhibitor (Fig. 7L−M). These results indicate that TAB2 is a target of TRIM27, and TRIM27 induces the degradation of TAB2 through the lysosomal degradation pathway.

### TRIM27 prevents TRAF6 autoubiquitination

**Fig. 8:**
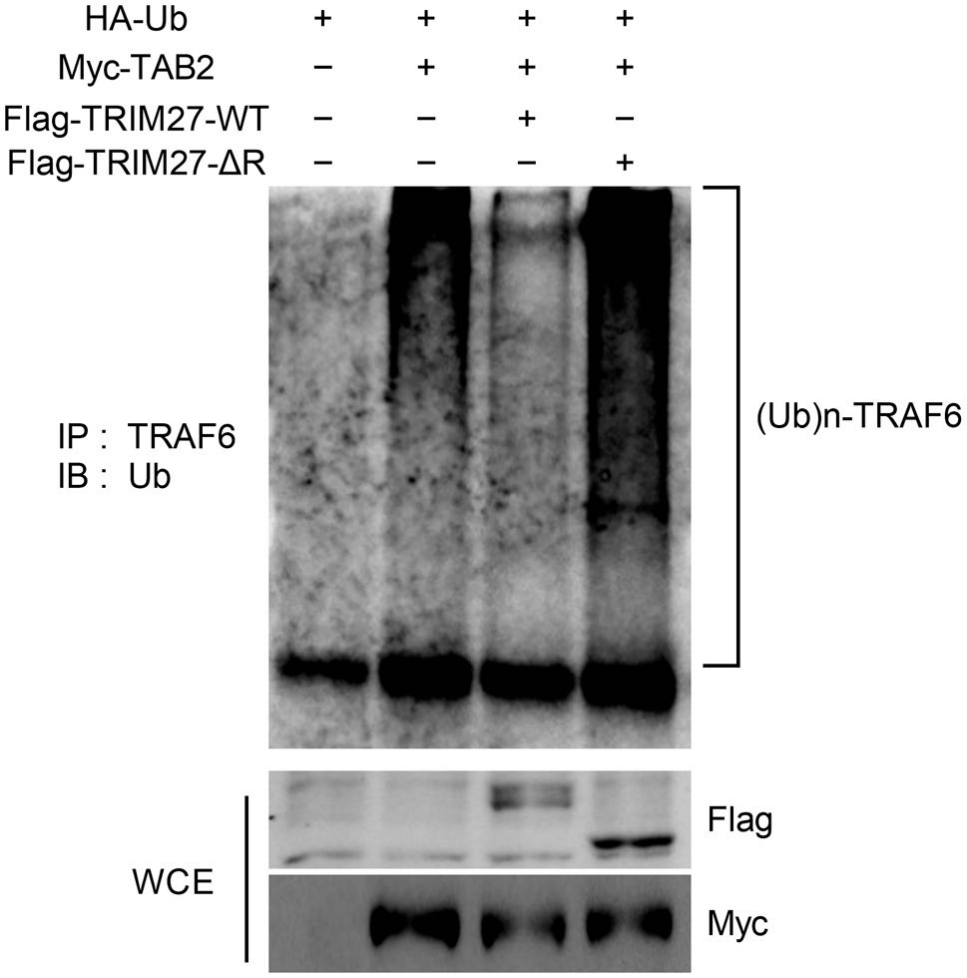
TRIM27 prevents TRAF6 autoubiquitination. 293T cells were transfected with HA-Ub and Myc-TAB2 in the presence or absence of Flag-TRIM27-WT or Flag-TRIM27-ΔR. Following a 36-hour incubation period, coimmunoprecipitation and immunoblot analyses were performed using the indicated antibodies

It has been documented that TAB2 facilitates TRAF6 autoubiquitination and thus contributes to NF-κB activation (Kishida et al. 2005; Walsh et al. 2008). TRAF6 is also a RING domain ubiquitin ligase that binds preferentially to K63-linked polyubiquitination chains and forms a complex with the TAK1 protein kinase and two adaptor proteins, TAB1 and 2 (Takaesu et al. 2000). Therefore, we investigated whether TRIM27, which targets TAB2, might interfere with TRAF6 autoubiquitination. When we transfected Myc-TAB2 and hemagglutinin (HA)-ubiquitin or TRIM27-WT or TRIM27-ΔR together into 293T cells, we detected autoubiquitination of TRAF6. We observed that TRIM27-WT reduced TRAF6 autoubiquitination. However, no effect was observed when the TRIM27-ΔR mutant was coexpressed with TRAF6 (Fig. 8). Since the formation of TRAF6 oligomers is necessary for its autoubiquitination (Hu et al. 2017; Yang et al. 2004), we examined the possibility that TRIM27 would affect the formation of TRAF6 oligomers. We detected the formation of TRAF6 oligomers by expressing and coimmunoprecipitating two distinctly tagged TRAF6 proteins, HA-TRAF6 and Flag-TRAF6; we found the formation of TRAF6 oligomers when it was overexpressed in 293T cells. We found that the expression of TRIM27 did not interfere with the formation of TRAF6 oligomers (Supplementary figure S1). Therefore, this indicated that TRIM27 inhibited NF-κB activation by suppressing TRAF6 autoubiquitination.

## Discussion

Our study revealed that TRIM27 is critical in osteoclast and osteoblast differentiation. Our observation showed that *Trim27*–deficient mice had reduced bone mineral density (Fig. 1). TRIM27 overexpression in osteoclasts suppressed RANKL-induced osteoclast differentiation (Fig. 3), whereas TRIM27 overexpression in osteoblast precursors enhanced osteogenesis (Fig. 5). These results demonstrate that TRIM27 is involved in the regulation of osteoclast and osteoblast differentiation, which is crucial for maintaining bone homeostasis.

NF-κB is crucial in regulating bone homeostasis in osteoclasts and osteoblasts, leading to increased bone resorption and decreased bone formation during the bone remodeling process (Boyce et al. 2010, 2015; Gilbert et al. 2000; Lee et al. 2015; Novack 2011). Furthermore, TAB2 functions as an adapter protein in TAK1 activation by linking TAK1 to TRAF6 in osteoclasts (Kishida et al. 2005; Walsh et al. 2008). Indeed, numerous TRIM proteins have been reported to be regulated by NF-κB regulatory cytokines and can regulate a series of processes in cells by promoting or inhibiting NF-κB signaling pathways by targeting different proteins through different manners of ubiquitination (Li et al. 2011; Qiu et al. 2013; Shi et al. 2014; Suzuki et al. 2016; Tomar and Singh 2014). Previous studies showed that TRIM27 interacts with IκB kinase (IKK) family members and TRAF family member-associated NF-κB activator binding kinase 1 (TBK1), which inhibits NF-κB-dependent and IRF3-dependent transcriptional activation (Zha et al. 2006). TRIM27 inhibits the type I IFN response against hepatitis C virus infection by inhibiting the IRF3 and NF-κB pathways (Zheng et al. 2019). Additionally, TRIM27 inhibits the pulmonary inflammatory response and apoptosis upon LPS by targeting the TLR4/NF-κB pathway (Wang et al. 2022). Through gain- or loss-of-function experiments with TRIM27, we observed that TRIM27 regulates NF-κB activity through degradation of IκB (Fig. 6). Therefore, our findings suggest that TRIM27 plays a pivotal role in bone remodeling by regulating osteoclast and osteoblast differentiation through the NF-κB pathway. Furthermore, we demonstrated that TRIM27 promotes TAB2 degradation through a lysosomal-dependent pathway, inhibiting the NF-κB pathway (Fig. 7). A recent report showed that TRIM27 plays a crucial role in regulating of hepatic ischemia/reperfusion injury by mediating the degradation of TAB2/3 and suppressing downstream TAK1–JNK/p38 signaling (Chen et al. 2021). Overall, these studies support our finding that TRIM27 can interact with TAB2 and mediates TAB2 degradation via the ubiquitin degradation pathway in bone cells, thereby regulating NF-κB signaling pathway.

Muscles and bones have a close and interdependent relationship, which is essential for maintaining overall musculoskeletal health. The age-related loss of skeletal muscle mass and strength is characteristic of sarcopenia and tends to occur approximately simultaneously (Ji et al. 2015; Mi et al. 2024). For example, the capacity of muscles to generate force declines with age, and the anabolic response of bone to muscle-derived stimuli also appears to be altered with age. Among TRIM family proteins, TRIM63 has been reported as a muscle-specific E3 ubiquitin ligase that plays a role in both muscle and bone. *TRIM63* knockout mice suppress denervation-induced muscle atrophy (Bodine et al. 2001), and a deficiency in *TRIM63* slightly reduces the cancellous bone mass in normal loaded conditions while suppressing the unloading-induced reduction in bone mass (Kondo et al. 2011). Similarly, TRIM27, another muscle-specific E3 ubiquitin ligase, induces muscle atrophy through ubiquitination-mediated degradation pathways, while sciatic nerve injury-induced muscle atrophy is attenuated in TRIM27 knockout mice (Joung et al. 2014). Like TRIM63, we expected that TRIM27 might also play an important role in osteoporosis or sarcopenia, skeletal diseases characterized by the loss of bone and muscle mass. However, contrary to expectations, reduced bone mineral density was observed in TRIM27 knockout mice (Fig. 1). Due to the known impact of muscle atrophy on bone metabolism and strength, we assessed the mRNA expression levels of *Trim27* in the bone and muscle tissues at different stages of aging in mice. In bone tissue, we observed an increase in the expression of *tartrate-resistant acid phosphatase (Trap/Acp5)*, an osteoclast marker, in aged mice, while the expression of *bone gamma-carboxyglutamate protein (Bglap/osteocalcin)*, an osteoblast marker, was downregulated in the bones of older mice compared to younger ones. Additionally, we observed increased muscle atrophy markers *Trim63* and *Artogin1* expressions in muscle tissue in aged mice. Interestingly, we observed that *Trim27* expression is upregulated in muscle tissue during age-induced muscle atrophy, whereas its expression is reduced in the bone tissue of older mice compared to younger mice (Supplementary figure S2). This differential expression pattern in muscle and bone suggests that TRIM27 may have a potential dual role in maintaining the function of these tissues in mice. However, the exact molecular pathways and interactions involved in this differential expression are fully understood. The relationship between muscle and bone may be regulated by molecular communication, involving various soluble factors under both physiological and pathological conditions (DiGirolamo et al. 2013). Therefore, our results suggest that the role of TRIM27 may involve direct regulation of bone rather than physiological regulation of bone by muscle. Furthermore, further studies are required to elucidate these mechanisms and their implications for tissue-specific functions under various conditions, including aging and disease.

## Conclusions

Our study elucidates the molecular mechanisms through which TRIM27 modulates NF-κB signaling in bone remodeling. These findings indicate that investigating the molecular pathways through which TRIM27 regulates osteoclast and osteoblast activity can provide insights into therapeutic strategies for preventing bone loss. Furthermore, we propose that pharmacological approaches targeting TRIM27 may also contribute to the development of treatments for musculoskeletal diseases.

## Electronic supplementary material

Below is the link to the electronic supplementary material.

Supplementary Material 1

Supplementary Material 2

Supplementary Material 3

## Data Availability

No datasets were generated or analysed during the current study.

## Abbreviations

TRIM27: Tripartite motif-containing 27
TAB2: TAK1-associated adapter protein 2
RANKL: Receptor activator of nuclear factor kappa B ligand
M: CSF-Macrophage colony-stimulating factor
BMP: 2-Bone morphogenetic protein-2
BMMs: Bone marrow macrophages
TRAP: Tartrate-resistant acid phosphatase
ALP: Alkaline phosphatase
PBS: Phosphate-buffered saline
BGLAP: Bone gamma-carboxyglutamate protein
NFATc1: Nuclear factor of activated T-cells cytoplasmic 1
OSCAR: Osteoclast-Associated Receptor
BSP: Bone sialoprotein
TRAF6: TNF Receptor Associated Factor 6
H&E: Hematoxylin and eosin
BV/TV: Bone volume per tissue volume
Tb.N: Trabecular number
Tb.Th: Trabecular thickness
Tb.Sp: Trabecular bone separation
Oc.N/BS: Osteoclast number per bone surface
Ob. N/BS: Osteoblast number per bone surface
