# Supplementary figures and images for: Tripartite motif-containing 27 negatively regulates NF-κB activation in bone remodeling
Source: Mol Med. 2025 Apr 18;31:141. doi: 10.1186/s10020-025-01204-7 (PMC12008848; doi:10.1186/s10020-025-01204-7)

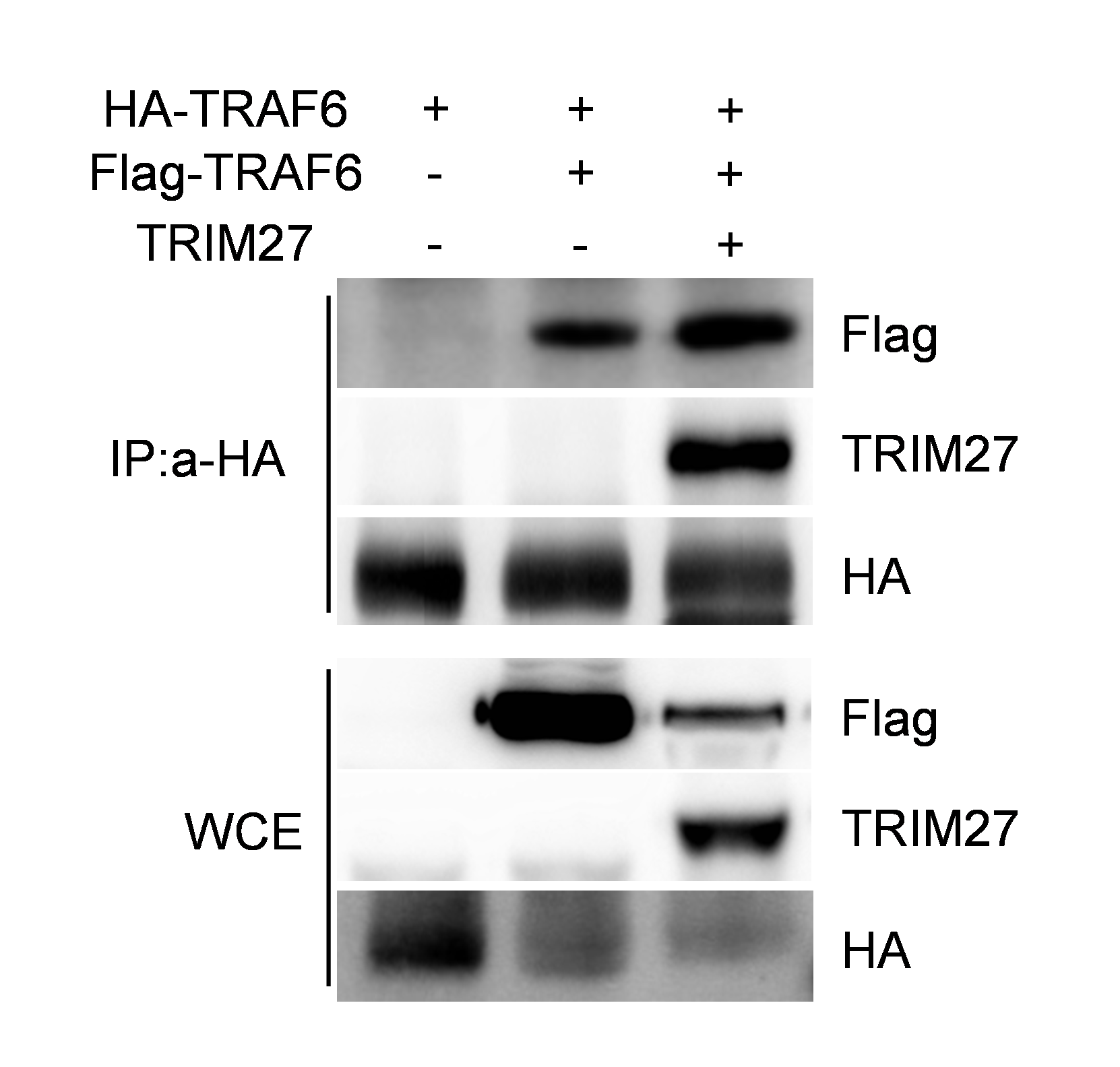

Supplement: Supplementary file 1 — Supplementary Material 1 [file 10020_2025_1204_MOESM1_ESM.tif]

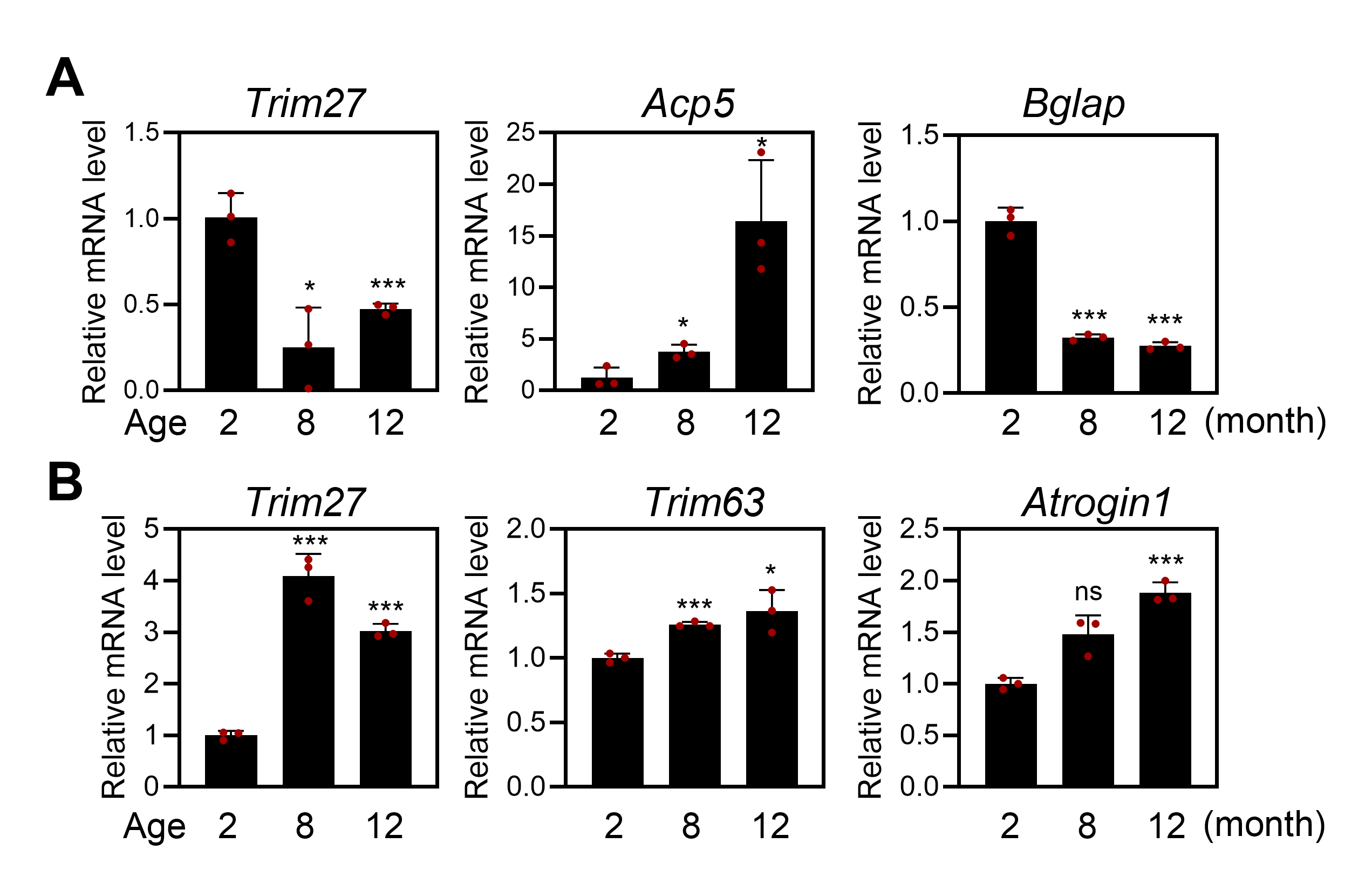

Supplement: Supplementary file 2 — Supplementary Material 2 [file 10020_2025_1204_MOESM2_ESM.tif]
