# Supplementary material for: Tripartite motif-containing 27 negatively regulates NF-κB activation in bone remodeling
Source: Mol Med. 2025 Apr 18;31:141. doi: 10.1186/s10020-025-01204-7 (PMC12008848; doi:10.1186/s10020-025-01204-7)
Supplement: Supplementary file 3 — Supplementary Material 3 [file 10020_2025_1204_MOESM3_ESM.docx]

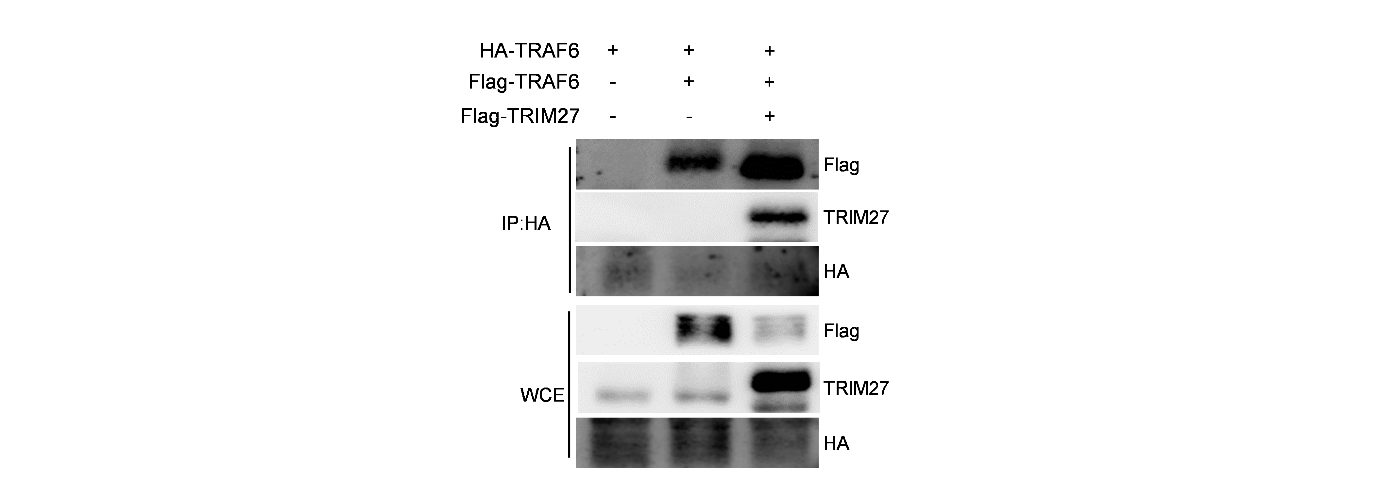


**Supplementary figure S1.** TRIM27 did not interfere with the formation of TRAF6 oligomers. 293T cells were transfected with HA-TRAF6 and/or Flag-TRAF6 or Myc-TRIM27. Following a 36-hour incubation period, coimmunoprecipitation and immunoblot analyses were performed using the indicated antibodies.

**
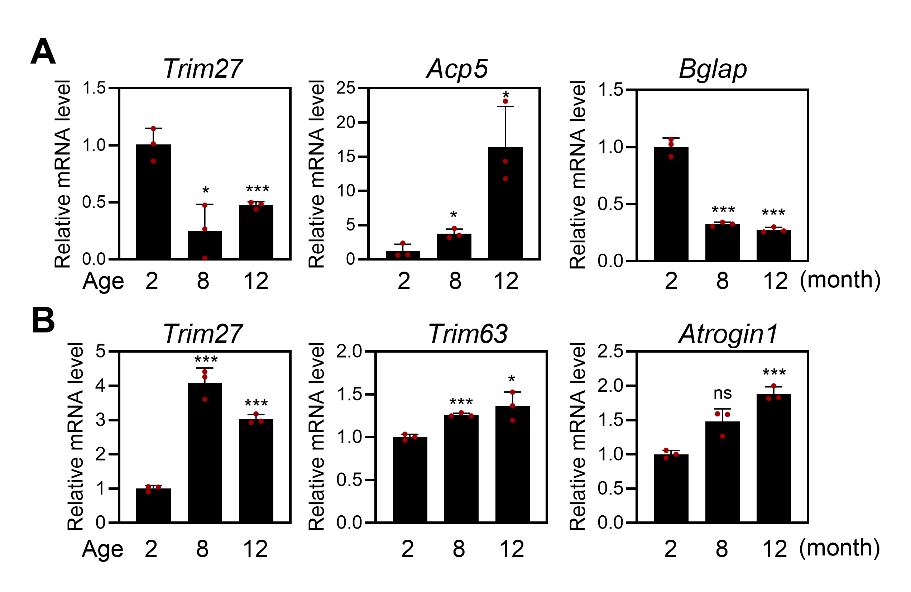
**

**Supplementary figure S2.** TRIM27 expression patterns in bone and muscle at different stages of aging in mice. (A) Total RNA was isolated from mouse bones, and *Trim27*, *Acp5*, and *Bglap* mRNA expression was assessed using quantitative real-time PCR. The presented values have been normalized to *Gapdh* levels. (B) Quantitative real-time PCR was used to assess mRNA expression of *Trim27*, *Trim63*, and *Atrogin1* in total RNA isolated from mouse skeletal muscle. The values shown are normalized to *Gapdh* levels. Data are presented as the mean ± SD of triplicate samples. **p* < 0.05, ****p* < 0.005 vs. 2 month. ns, not significant (*p* > 0.05).
